# Early outcomes of a community‐based participatory research (CBPR) approach to investigating brain health and ADRD phenotypes following traumatic brain injury

**DOI:** 10.1002/alz.093578

**Published:** 2025-01-09

**Authors:** Kristen Dams‐O'Connor

**Affiliations:** ^1^ Icahn School of Medicine at Mount Sinai, New York, NY, USA; One Gustave Levy Place, New York, NY USA

## Abstract

This session is proposed for consideration to be presented as part of the Partnering with Research Participants Professional Interest Area (PIA), for AAIC 2024 FRS titled *Participant and Public Involvement Across Alzheimer’s Disease Research Studies: Conceptual and Empirical Strategies for Equitable Participation*. This session will focus on areas of opportunity in the involvement of research volunteers to further and enhance research design and practices in different aspects of AD and ADRD research studies. This session is specifically focused on conceptual and applied approaches for the involvement of research volunteers across all study designs. We will use a use‐case example to illustrate the implementation of a DoD CDMRP‐funded Focused Program Award designed to investigate brain health and ADRD phenotypes following traumatic brain injury. The ENRICH brain health study is founded in a community‐based participatory research (CBPR) approach, with one of the five studies focused on the formation and implementation of a Community Advisory Group for Brain Health (CAGE‐BH). The CAGE‐BH consists of individuals with lived experience of brain injury, neurodegenerative disease, military service history, leadership of or participation in local or national brain injury or dementia care advocacy groups, and veteran service organizations. The CAGE‐BH is involved in the development and implementation at all stages of the four research studies comprising the ENRICH Focused Program Award. The proposed session will share early outcomes of a community‐based participatory research (CBPR) approach to investigating brain health and ADRD phenotypes following traumatic brain injury.

